# The Hypervirulent Strain of Clostridium Difficile: NAP1/B1/027 - A Brief Overview

**DOI:** 10.7759/cureus.3977

**Published:** 2019-01-29

**Authors:** Rawish Fatima, Muhammad Aziz

**Affiliations:** 1 Internal Medicine, Dow University of Health Sciences, Karachi, PAK; 2 Internal Medicine, University of Kansas Medical Center, Kansas City, USA

**Keywords:** clostridium difficile, diarrhea, ribotype 027, recurrence, nap1/b1/027

## Abstract

Clostridium difficile is a gram-positive bacterium notorious for causing epidemic diarrhea globally with a significant health burden. The pathogen is clinically challenging with increasing antibiotic resistance and recurrence rate. We provide here an in-depth review of one particular strain/ribotype 027, commonly known as NAP1/B1/027 or North American pulsed-field gel electrophoresis type 1, restriction endonuclease analysis type B1, polymerase chain reaction ribotype 027, which has shown a much higher recurrence rate than other strains.

## Introduction and background

Clostridium difficile (C. diff) is a gram-positive, anaerobic, motile, spore-forming, rod-shaped bacteria [1-2]. It has been isolated from almost all mammals, including pigs, cows, horses, elephants, and Kodiak bears, as well as in poultry and ostriches. It has also been found in the soil and feces of humans and animals. It is transmitted from person to person by the fecal-oral route. The C. diff isolates found in animals are similar to the ones found in humans, but according to Hensgens et al., this similarity does not mean that interspecies transmission occurs. However, immunocompromised people are still at risk for interspecies transmission [1]. Its pathogenicity is dependent on the two toxins that it produces: enterotoxin A (Toxin A or TcdA) and cytotoxin B (Toxin B or TcdB). Enterotoxin damages the actin in target cells which leads to neutrophil infiltration, inflammation, and necrosis of epithelial cells. Cytotoxin B has been shown to damage tight junctions of epithelial cells, which increases vascular permeability and causes hemorrhage [2-3]. These toxins form the basis of stool analysis when diagnosing people with the suspected infection. Despite all the virulence characters described, C. diff is a poor competitor against other gut flora in the human colon. In a healthy colon, this pathogen is not in sufficient quantity to produce a clinically significant disease. Risk factors that disrupt this balance include antibiotics exposure, health care environment, acid suppressants, and elemental diet. The bacterium can cause severe watery diarrhea that can progress to pseudomembranous colitis [3-8]. It has been named as one of the three microorganisms with an ‘urgent’ threat level by the Centers for Disease Control and Prevention (CDC) based on its public health impact in the United States (US) with an estimated $1.5 billion US in annual health care expenditures [8]. Patients who have more than three episodes of unexplained and new onset unformed stools in 24 hours should be referred for testing for a Clostridium difficile infection (CDI). Also, patients with risk factors described previously should undergo testing for this pathogen [9]. The ribotype 027 strain of C. diff is particularly noteworthy as contradicting evidence in the literature is present regarding the disease severity it causes. We provide here a brief overview of the epidemiology, pathophysiology, and treatment of this particular strain. 

## Review

Ribotypes and prevalence of Clostridium difficile (C. diff)

Clostridium difficile can be characterized according to its ribotyping which is performed using the polymerase chain reaction. Several different ribotypes have been associated with CDI. The ribotypes 001, 002, 014, 046, 078, 126, and 140 have been found to be prevalent in the Middle East [10-12]. In Asia, ribotypes 001, 002, 014, 017, and 018 are more prevalent [13-15]. The predominant strains in Europe and North America include ribotypes 001, 014, 020, 027, and 078 [6]. The ribotype 027 (also referred to as NAP1/B1/027) has emerged in the last decade. Studies have underlined antimicrobial resistance as one of the causes of its epidemic outbreaks. Capillary electrophoresis (CE) ribotyping is used as the standard for characterization of C. diff isolates. This method relies on the intergeneric region variability between 16S and 23S ribosomal deoxyribonucleic acid (DNA) [16]. Ribotype 027 was found to have reduced susceptibility to metronidazole, rifampicin, moxifloxacin, clindamycin, imipenem, and chloramphenicol [17-18]. It is clinically and financially concerning as it leads to severe disease presentation, as well as antimicrobial resistance with high morbidity and mortality rates as compared to other strains [19]. Strains, such as ribotype 027 (especially its spores), spread more easily within the hospital because they can resist the hospital environment, cleaning, and disinfectants [1]. An observational study conducted on patients admitted with diarrhea in a Veteran Affairs Medical Center showed that around 22% of the patients were positive for the NAP1/B1/027 strain out of all the people who tested positive for CDI. Further, a reduction in the rate of diarrhea caused by the NAP1/B1/027 strain was observed with a prevalence of 16.9% in 2016, down from 26.2% in 2013. An increase in the level of awareness and education was thought to be the reason for this decline [20]. The prevalence of this strain in North America is reportedly around 22% - 36%. Ribotype 027 was identified as the most prevalent strain causing CDI with recent outbreaks in North America [20-22]. The prevalence of this strain was shown to be 48% in hospitals in Poland with an outbreak of CDI during September 2011 to August 2013 [21].

NAP1/B1/027 strain

Toxigenicity and Pathogenesis

The North American pulsed-field gel electrophoresis type 1, restriction endonuclease analysis type B1, polymerase chain reaction ribotype 027 (NAP1/B1/027) strain has been shown to contain a gene locus, CdtLoc, that encodes for CD196 ADP-ribosyltransferase (CDT) or binary toxin. The bacterium also produces Toxin A and Toxin B, similar to non-027 ribotypes, through the PaLoc gene locus [23-24]. CDT was first isolated by Popoff et al. [25]. The toxin comprises two separate toxin components: CDTa and CDTb. CDTa, which is an ADP-ribosyltransferase enzyme, modifies actin which results in depolymerization and destruction of the actin cytoskeleton in the gut. CDTb binds to gut cells and increases uptake of CDTa. The destruction caused by CDT favors adherence of bacteria and increased uptake of Toxin A and Toxin B [26].

In addition to the toxins, this strain (along with few others) carries a base pair frameshift deletion at nucleotide 117 of the TcdC gene, which is a negative regulator of Toxins A and B. A mutation in this gene thus causes hyperexpression of toxins by this particular strain. Warny et al. showed that NAP1/B1/027 produces Toxin A approximately 16 times and Toxin B approximately 23 times more than the control strains [27]. One study also proposed that increased sporulation by this strain may also be associated with the increased spread of CDI [28]. The virulent factors associated with NAP1/B1/027 strain have been summarized in Table 1.

**Table 1:** Virulent factors associated with NAP1/B1/027 strain CDTa toxin: CD196 ADP-ribosyltransferase a toxin; CDTb toxin: CD196 ADP-ribosyltransferase b toxin; NAP1/B1/027: North American pulsed-field gel electrophoresis type 1, restriction endonuclease analysis type B1, polymerase chain reaction ribotype 027

	Virulent factor	Mechanism
1.	Toxin A (Enterotoxin A or TcdA)	Damages the actin in target cells which leads to neutrophil infiltration, inflammation, and necrosis of epithelial cells [24].
2.	Toxin B (Cytotoxin B or TcdB)	Damages tight junctions of epithelial cells, which increases vascular permeability and causes hemorrhage [24].
3.	CDTa toxin	Modification of actin with ADP-ribosylation that results in actin depolymerization and destruction of the cytoskeleton that assists in adherence of bacteria to gut epithelial cells [25-26].
4.	CDTb toxin	Facilitates uptake of CDTa toxin into the gut epithelial lining [25-26].
5.	Hypersporulation	Increases reproduction and spread of bacteria [28].
6.	TcdC gene mutation (18-bp deletion)	Increases the production of Toxin A and Toxin B by down-regulation of feedback inhibitor involved in suppressing toxin production [27].

Previous studies have shown contradicting evidence regarding the severity of disease caused by this particular strain. A recent retrospective analysis by Bauer et al. concluded that NAP1/B1/027 was associated with a decreased odds of severe disease (odds ratio (OR): 0.35, 95% confidence interval (CI) 0.13 - 0.93) and did not increase in-hospital mortality (OR: 1.02, 95% CI 0.53 - 1.96) or recurrence rate (OR: 1.16, 95% CI 0.36 - 3.77) [23]. Several other studies conducted (including cross-sectional, case-control, and cohort studies) did not show any worse outcomes compared to other strains [29-31]. Sirad et al. demonstrated that although NAP1/B1/027 strain may produce more toxins compared to other strains, they produced fewer spores and were not always associated with severe disease [32]. On the contrary, Rao et al. conducted a cohort study and concluded that ribotype 027 was associated with severe CDI (OR: 1.73, 95% CI 1.03 - 2.89; p = 0.037) and increased mortality (OR: 2.02, 95% CI 1.19 - 3.43; p = 0.009) compared to other ribotypes [24]. Another study showed similar results with the North American pulsed-field gel electrophoresis type 1 (NAP1) strain. Multivariate regression analysis exhibited an increase in the severity of CDI with the NAP1 strain (OR: 1.66, 95% CI: 1.90 - 2.54) and increased mortality (OR: 2.12, 95% CI: 1.22 - 3.68) [33]. One study from Quebec labeled this strain to be responsible for severe diseases twice as frequently as compared to other strains [34].

The basis for these contradictory findings can be explained by several reasons, including study design, study population, sample size, the method of detection for C. diff, study setting, and unmeasured confounders. Given these contradictory results, healthcare providers should focus on treating this infection based on their clinical judgment and markers of severe infection, including the number of diarrheal episodes, signs of dehydration, creatinine level, albumin level, white blood cell count, associated co-morbidities, immunocompromised state, etc.

Prevention

Preventive strategies employed for NAP1/B1/027 strain are similar to strategies taken for other strains. These include barrier methods (gloves and gown while examining patient), use of disposable equipment, handwashing with soap and water, disinfecting the environment, and antimicrobial stewardship [35]. Further vaccines are being developed targeting the toxins, including TcdA and TcdB, for simultaneous prevention and treatment of CDI. Actoxumab and bezlotoxumab, which are monoclonal antibodies against TcdA and TcdB, are being investigated for this purpose. A combined Phase III trial (MODIFY I (NCT01241552) and MODIFY II (NCT01513239)) showed benefit from bezlotoxumab, but the combination of actoxumab and bezlotoxumab did not yield any further benefit [36]. Bezlotoxumab has received Food and Drug Administration (FDA) approval in October 2016 and is to be used in patients more than 18 years of age, who are at high risk of recurrence from CDI, and are receiving antibiotics [37]. A novel tetravalent vaccine against TcdA, TcdB, CDTa, and CDTb has been proposed by Secore et al. using a hamster model which has shown promising results [38].

A novel drug, SYN-004 (ribaxamase), is under investigation that has shown promising results for preventing CDI. This drug, which is a β-lactamase, is excreted into the gut and degrades the excess antibiotic that prevents disruption of normal gut flora, ultimately preventing CDI [39]. The Phase IIa clinical trial of this drug showed that ribaxamase at a dose of 150 mg every six hours results in an undetectable concentration of ceftriaxone in the intestine which can potentially decrease the likelihood of a C. diff infection, given the less probability of disruption of the gut bacteria. 

Resistance to Antibiotics and Treatment

Cases of NAP1/B1/027 reported in Panama were found to be highly resistant to clindamycin, moxifloxacin, levofloxacin, ciprofloxacin, and rifampin but were susceptible to metronidazole and vancomycin [40]. Susceptibility of ribotype 027 and non-027 ribotypes to different antibiotics was tested in a study in Canada. Ribotype 027 showed a resistance of 92.2% to moxifloxacin compared to 11.2% for other strains. Similarly, 78.2% of ribotype 027 strains were resistant to ceftriaxone compared to 15.7% of other strains. Ribotype 027 demonstrated a greater than four-fold higher minimum inhibitory concentration (MIC) to metronidazole (4 vs. 1 μg/ml) and two-fold higher MIC for fidaxomicin (1 vs. 2 μg/ml). For clindamycin and vancomycin, the resistance was similar in both groups [41].

Resistance to erythromycin is linked to mutations in the ribosomal methylase genes, whereas resistance to fluoroquinolones is due to a mutation in DNA gyrase. Resistance to rifamycin and fidaxomicin is attributed to ribonucleic acid (RNA) polymerase methylation. The presence of phenicol and lincosamide genes has been shown to cause resistance to linezolid. A study conducted in hospitals of Mexico showed some isolates of ribotype 027 to have reduced susceptibility to fidaxomicin despite the unavailability of this drug in Mexico and the patients being unexposed to it [42]. Antibiotics form the basis of treatment for the NAP1/B1/027 strain. Currently, no specific Infectious Diseases Society of America (IDSA) guidelines are available to guide treatment for this particular strain, and hence, the treatment is similar to a non-NAP1/B1/027 strain [9]. Based on the current guidelines for treating CDI overall, we propose the following table for treating infection caused by the NAP1/B1/027 strain (Table 2).

**Table 2:** Proposed Treatment for NAP1/B1/027 Strain IV: intravenous; IVIG: intravenous immunoglobulin; NAP1/BI/027: North American pulsed-field gel electrophoresis type 1, restriction endonuclease analysis type B1, polymerase chain reaction ribotype 027

	First line treatment	Alternative treatment
Initial non-severe infection	Oral vancomycin, 125 mg four times daily for 10 days	Fidaxomicin, 200 mg twice daily for 10 days; If neither is available, then use metronidazole, 500 mg three times daily for 10 days
First non-severe recurrence	Repeat oral vancomycin, 125 mg four times daily for 10 days	Fidaxomicin, 200 mg twice daily for 10 days
Second non-severe recurrence	Oral vancomycin taper as follow: 125 mg four times daily for seven to 14 days, 125 mg twice daily for seven days, 125 mg twice once daily for seven days, 125 mg once every other day for seven days, 125 mg once every three days for 14 days	Fidaxomicin, 200 mg orally twice daily for 10 days, or a fecal microbiota transplant
Subsequent non-severe recurrence	Fecal microbiota transplant	Tapering oral vancomycin with probiotics, IVIG, fidaxomicin
Severe disease	Oral vancomycin, 125 mg four times daily, increase to 500 mg four times daily if no improvement noted in 24-48 hours or associated complications, including renal failure, ileus, etc.	Fidaxomicin if the patient cannot tolerate oral vancomycin for any reason
Ileus	Add IV metronidazole, 500 mg every eight hours, to oral vancomycin or fidaxomicin therapy; consider general surgery consult as needed	Intracolonic vancomycin, IVIG

This strain has not shown any resistance to fidaxomicin, but there has been some contradicting evidence to this. A case report was published in 2017 in which the NAP1 C. diff infection, resistant to treatment with fidaxomicin and fecal transplants, was effectively treated with intravenous immunoglobulin (IVIG) [43]. Given the emerging threat of antibiotic resistance, increasing awareness, controlling infections, and antimicrobial stewardship can be effective measures to reduce this threat [17].

Currently, several novel antibiotics are under investigation which have gone through various randomized controlled trials for CDI treatment. Ridinilazole and cadazolid have completed Phase II trials, while surotomycin has completed two Phase III trials which have shown promising results [44-47]. 

## Conclusions

The data regarding the NAP1/B1/027 strain is inconclusive with ongoing debates whether this particular strain is associated with severe disease. Further research, including meta-analyses, are needed to solve this enigma. Clinicians should guide treatment based on their judgment and objective evidence of disease severity. 
